# Epilepsy is an important feature of KBG syndrome associated with poorer developmental outcome

**DOI:** 10.1002/epi4.12799

**Published:** 2023-08-18

**Authors:** Nathan Buijsse, Floor E. Jansen, Charlotte W. Ockeloen, Marjan J. A. van Kempen, Shimriet Zeidler, Marjolein H. Willemsen, Emanuela Scarano, Sonia Monticone, Evelien Zonneveld‐Huijssoon, Karen J. Low, Allan Bayat, Sanjay M. Sisodiya, Debopam Samanta, Gaetan Lesca, Danielle de Jong, Jaqcues C. Giltay, Nienke E. Verbeek, Tjitske Kleefstra, Eva H. Brilstra, Danique R. M. Vlaskamp

**Affiliations:** ^1^ Department of Medical Genetics University Medical Center Utrecht Utrecht The Netherlands; ^2^ Department of Pediatric Neurology, Brain Center University Medical Center Utrecht Utrecht The Netherlands; ^3^ Department of Human Genetics Radboud University Medical Center Nijmegen The Netherlands; ^4^ Department of Clinical Genetics Erasmus Medical Center Rotterdam The Netherlands; ^5^ Department of Pediatrics St. Orsola‐Malpighi Hospital Bologna Italy; ^6^ Department of Pediatrics Azienda Ospedaliero Universitaria Maggiore della Carità Novara Italy; ^7^ Department of Genetics, University of Groningen University Medical Center Groningen Groningen The Netherlands; ^8^ Department of Clinical Genetics, University Hospitals Bristol and Weston NHS trust University of Bristol Bristol UK; ^9^ Department for Genetics and Personalized Medicine Danish Epilepsy Centre Dianalund Denmark; ^10^ Institute for Regional Health Services University of Southern Denmark Odense Denmark; ^11^ Department of Clinical and Experimental Epilepsy UCL Queen Square Institute of Neurology and Chalfont Centre for Epilepsy Chalfont St Peter UK; ^12^ Child Neurology Section, Department of Pediatrics University of Arkansas for Medical Sciences Little Rock Arkansas USA; ^13^ Department of Genetics University Hospitals of Lyon Lyon France; ^14^ Department of Neurology Academic Center for Epileptology Kempenhaeghe/MUMC+ Heeze The Netherlands; ^15^ Department of Pediatrics University Medical Center Utrecht Utrecht The Netherlands

**Keywords:** *ANKRD11 gene*, genotype–phenotype correlation, neurodevelopment, seizure

## Abstract

**Objective:**

The aim of this study was to describe the epilepsy phenotype in a large international cohort of patients with KBG syndrome and to study a possible genotype–phenotype correlation.

**Methods:**

We collected data on patients with *ANKRD11* variants by contacting University Medical Centers in the Netherlands, an international network of collaborating clinicians, and study groups who previously published about KBG syndrome. All patients with a likely pathogenic or pathogenic *ANKRD11* variant were included in our patient cohort and categorized into an “epilepsy group” or “non‐epilepsy group”. Additionally, we included previously reported patients with (likely) pathogenic *ANKRD11* variants and epilepsy from the literature.

**Results:**

We included 75 patients with KBG syndrome of whom 26 had epilepsy. Those with epilepsy more often had moderate to severe intellectual disability (42.3% vs 9.1%, RR 4.6 [95% CI 1.7–13.1]). Seizure onset in patients with KBG syndrome occurred at a median age of 4 years (range 12 months – 20 years), and the majority had generalized onset seizures (57.7%) with tonic–clonic seizures being most common (23.1%). The epilepsy type was mostly classified as generalized (42.9%) or combined generalized and focal (42.9%), not fulfilling the criteria of an electroclinical syndrome diagnosis. Half of the epilepsy patients (50.0%) were seizure free on anti‐seizure medication (ASM) for at least 1 year at the time of last assessment, but 26.9% of patients had drug‐resistant epilepsy (failure of ≥2 ASM). No genotype–phenotype correlation could be identified for the presence of epilepsy or epilepsy characteristics.

**Significance:**

Epilepsy in KBG syndrome most often presents as a generalized or combined focal and generalized type. No distinctive epilepsy syndrome could be identified. Patients with KBG syndrome and epilepsy had a significantly poorer neurodevelopmental outcome compared with those without epilepsy. Clinicians should consider KBG syndrome as a causal etiology of epilepsy and be aware of the poorer neurodevelopmental outcome in individuals with epilepsy.

**Key points**

- Seizures occurred at a median age of 4 years, with generalized seizures being most prevalent.
- No electroclinical syndrome specific for KBG syndrome was identified, but overlap with other (combined focal and) generalized epilepsy syndromes was seen.
- Patients with KBG syndrome and epilepsy have significantly more severe intellectual disability, compared with those without epilepsy.
- Seizure freedom (≥1 year without seizures) was reached in half of the patients; however, 27% of patients had drug‐resistant epilepsy (failure of ≥2 ASM).
- No genotype–phenotype correlation was seen for the presence of epilepsy or epilepsy characteristics.

## 1 INTRODUCTION

KBG syndrome (OMIM 148050) was first described by Herrman et al. in 1975 in three families with the surname initials K, B, and G.^1^ The affected patients are characterized by global developmental delay (DD), intellectual disability (ID), behavioral issues, short stature, macrodontia, facial dysmorphism, and multiple congenital anomalies.^1^, ^2^ More than 100 individuals with KBG syndrome have been reported since.^2^ No definite clinical criteria for diagnosing KBG syndrome have been established, but the criteria of Skjei et al. and Low et al. are most commonly used.^2^, ^3^, ^4^ According to Skjei et al., four of eight major criteria should be met for a clinical diagnosis.^3^ Low et al. suggest a diagnosis of KBG syndrome in patients with DD, speech impairment or behavioral issues with at least two major or one major and two minor criteria.^4^

A diagnosis of epilepsy—a major criterion according to Skjei et al. and a minor criterion according to Low et al.—has been reported in approximately 30% of patients in a systematic review of 140 patients with KBG syndrome.^5^ The onset of epilepsy is predominantly between infancy and mid‐teens and seizure remission occurred in the majority after adolescence with good response to anti‐seizure medication (ASM).^4^, ^6^ Heterogeneous seizure types have been reported, with tonic–clonic seizures, absences, myoclonic seizures, and unclassified sleep‐related seizures with motor symptoms being most common, but no specific epilepsy syndrome has been identified.^3^, ^4^, ^6^, ^7^, ^8^, ^9^

KBG syndrome is an autosomal dominant disorder caused by pathogenic variants in the ankyrin repeat domain‐containing protein 11 gene (*ANKRD11*) or chromosomal microdeletions in 16q24.3 including *ANKRD11*. ANKRD11 is part of a family of ankyrin repeat‐containing cofactors that play a role in epigenetic regulation of histone acetylation and deacetylation.^10^ It regulates dendritic differentiation and pyramidal neuron migration in the developing mouse cerebral cortex.^11^ *ANKRD11* encodes two functional repressor domains (RD1 and RD2), one activator domain (AD), and one ankyrin repeat domain.^10^

To date, a detailed classification of the seizures, epilepsy type, or syndrome was only given in 26 patients from 12 studies: only two patients had an epilepsy syndrome classification.^5^, ^6^, ^8^, ^9^, ^12^, ^13^, ^14^, ^15^, ^16^, ^17^, ^18^, ^19^ Only in two case series—describing 12 patients—were the epilepsy characteristics described as the main subject.^8^, ^15^ A genotype–phenotype correlation study suggested a more severe ID in patients in whom all functional domains or the last functional domain was disrupted,^20^ but no genotype–phenotype correlation studies have been performed for the presence and type of epilepsy in patients with KBG syndrome.

Our aim is to describe the epilepsy characteristics in a large international cohort of patients with (likely) pathogenic *ANKRD11* variants or deletions and to perform a genotype–phenotype analysis for the presence and type of epilepsy. Our patient cohort will be compared with historical cases identified by a literature review.

## 2 PATIENTS AND METHODS

### 2.1 Patient cohort

Patients data were collected between May 2020 and March 2021 by contacting all University Medical Centers in the Netherlands, personal communication with collaborating colleagues in Europe, and by contacting medical centers that previously published about KBG syndrome. For each center, one clinical geneticist or (pediatric) neurologist was responsible for the patient inclusion and data collection in their center. The single inclusion criterion was a confirmed genetic diagnosis of KBG syndrome based on a (likely) pathogenic variant in *ANKRD11* or a chromosomal 16q24.3 microdeletion including (part of) *ANKRD11*. To prevent duplicates, we asked whether patients had been included in a previous study. Included patients were classified into an “epilepsy group”—when having (a history of) epilepsy—or a “non‐epilepsy group”. Patients with a chromosome 16q24.3 microdeletion including *ANKRD11* among other genes were discussed as a separate group.

### 2.2 Genotyping

*ANKRD11* variants were all described with reference to transcript “NM_013275.6”. Chromosomal microdeletions were reported with reference to the Genome Reference Consortium Human Reference sequence version 37 (GRCh37/hg19). Pathogenicity of variants was determined in accordance with the American College of Medical Genetics and Genomics guidelines.^21^ For missense and splice‐site mutations, pathogenicity was predicted by using in silico prediction tools (Align GVGD, conserving scores, Grantham, SIFT, PolyPhen, NNsplice, MaxENT, and Splice Site Finder; see Table S1). We classified all nonsense variants, frameshift variants, and partial *ANKRD11* deletions as truncating variants.

### 2.3 Phenotyping

We collected data from medical records, genetic testing, electroencephalogram (EEG), and neuroimaging results of all patients. All EEG results were analyzed by a pediatric neurologist.

A diagnosis of epilepsy was made according to the International League Against Epilepsy (ILAE) 2014 definition.^22^ Seizure types, epilepsy types, and epilepsy syndromes were classified in accordance with the 2017 and 2022 ILAE classifications.^23^, ^24^, ^25^, ^26^, ^27^, ^28^ The epilepsy type was classified as “combined focal and generalized epilepsy” if seizures or EEG findings together showed features of both a focal and a generalized epilepsy. Seizure freedom was defined as having no seizures for at least 1 year and drug‐resistant epilepsy as failure of adequate trials of two antiepileptic drug schedules to achieve sustained seizure freedom, in accordance with the ILAE definition.^29^ Developmental delay was defined as present if delay was seen while children were under 5 years of age in at least two developmental domains (fine motor, gross motor, cognition, speech/language, personal/social, or activities of daily living), and functional age was at least 33% below the chronological age for this domain.^30^, ^31^ Intellectual disability was classified based on the intellectual quotient (IQ) as mild (IQ 50–69) or moderate to severe (IQ <50) or based on information on level of functioning in accordance with the DSM‐V.^31^ Motor impairment was defined as not walking independently at the time of assessment (and ≥24 months of age), and language impairment was defined as having no fluent speech at the time of assessment (and ≥4 years of age).

### 2.4 Genotype–phenotype correlation

Genotype–phenotype correlation was studied in patients with truncating variants. The presence of epilepsy and specific epilepsy characteristics, including (1) type of epilepsy; (2) prevalence of seizure freedom; and (3) prevalence of drug‐resistant epilepsy, were compared between patients with different disrupted functional domains.

### 2.5 Historical cohort

We performed a literature search in June 2022 using PubMed to identify previously reported patients with a diagnosis of KBG syndrome, confirmed by the identification of a (likely) pathogenic *ANKRD11* variant or a 16q24.3 microdeletion including *ANKRD11*, and a (former) diagnosis of epilepsy (see Figure S1 for the flowchart). If data about epilepsy characteristics were incomplete, we contacted the corresponding authors to collect additional data.

### 2.6 Data analysis

IBM SPSS statistics 26 was used for statistical analysis of our data. Baseline characteristics were analyzed using descriptive statistics. Differences in prevalence between groups (epilepsy vs no epilepsy group) were examined by Fisher's exact and Pearson's chi‐squared tests. Relative risks (RR) were calculated with subsequent 95% confidence intervals (CI). Logistic regression analysis was used to perform genotype–phenotype correlation analysis. *P*‐values were multiplied with the number of tests (n = 10) to correct for multiple testing (Bonferroni correction). *P*‐values <0.05 were considered statistically significant.

### 2.7 Standard protocol approvals and patient consents

This observational, retrospective study has been reviewed and approved by the Medical Ethical Committee of the University Medical Centre Utrecht (UMCU) and does not fall under the Medical Research Involving Human Subjects Act (reference: 20‐013/C). Informed consent for the study was given by all patients or—if patients were under 16 years of age or intellectually disabled—by their legal guardians.

## 3 RESULTS

### 3.1 Patient cohort (n = 75)

We identified 82 patients from participating University Medical Centers in the Netherlands (n = 63), and seven international medical centers in Italy (n = 12), the United Kingdom (n = 4), Denmark (n = 2), France (n = 1), and the USA (n = 1). We included 75 patients (91.4%) with a (likely) pathogenic *ANKRD11* variant or deletion in our patient cohort (see Table S1 for classification of pathogenicity). Four patients (4.8%) were excluded: three patients had a *ANKRD11* variant of unknown significance and for one patient the boundaries and size of the 16q24.3 microdeletion were unknown. Three other patients (3.7%) had a chromosome 16q24.3 microdeletion including *ANKRD11* among other genes and are described separately.

### 3.2 Phenotype in the patient cohort (n = 75)

The median age at last assessment was 12 years (range 23 months — 66 years; Table 1 and Table S2). Six patients (8%) were previously reported in literature.^9^, ^32^ Seventy‐one patients (94.7%) had a truncating *ANKRD11* variant (50 frameshift; 19 nonsense; 2 partial *ANKRD11* deletion); three (4.0%) a missense variant; and one (1.3%) a splice‐site variant. Inheritance was tested in 52 of 75 patients and variants occurred de novo in 46 of them (88.5%). Epilepsy was diagnosed in 26 patients (34.7%). The majority of patients had a global DD (86.6%), followed by ID in 54.3%. Most patients without ID had learning problems (34.3%). In a minority, autistic spectrum disorder (ASD, 24.3%) or attention deficit hyperactivity disorder (ADHD, 28.4%) was diagnosed. Brain imaging was available in 41 patients; anomalies were present in 41.5% of patients, with a Dandy–Walker complex malformation (n = 8) as most prevalent finding.

**TABLE 1:** Clinical features of our cohort and the historical cohort.

	Patient cohort (n = 75)	Subgroups patient cohort			Historical cohort (n = 37)
		Epilepsy group (n = 26)	Non‐epilepsy group (n = 49)	*P* value	
Male gender, n (%)	42/75 (56.0)	15/26 (57.7)	27/49 (55.1)		18/35 (48.6)
Median age at last assessment (range)	12 years 10 m	13 years 8 months	12 years		12 years
	(1 year 11 months–66 years)	(5 years–39 years)	(1 year 11 months –66 years)		(2 years–41 years)
**Genotype**					
Variant type					
Truncating, n (%)	71/75 (94.7)	25/26 (96.1)	46/49 (93.9)		35/37 (94.6)
Missense, n (%)	3/75 (4.0)	1/26 (3.8)	2/49 (4.1)		2/37 (5.4)
Splice site, n (%)	1/75 (1.3)	0/26 (−)	1/49 (2.0)		0/37 (−)
Inheritance of variant					
De Novo, n (%)	46/52 (88.5)	14/18 (77.8)	32/34 (94.1)		19/22 (86.4)
**Neurodevelopmental phenotype**					
Developmental delay, n (%)	65/75 (86.6)	24/26 (92.3)	41/49 (83.7)	1.00	31/32 (96.9)
Developmental plateauing or regression, n (%)	2/65 (3.1)	2/19 (10.5)	0/46 (−)	0.82	Reported in two patients
Cognition^a^					
Normal, n (%)	8/70 (11.4)	2/26 (7.7)	6/44 (13.6)	1.00	
Learning problems, n (%)	24/70 (34.3)	4/26 (15.4)	20/44 (45.5)	0.10	
ID, n (%)	38/70 (54.3)	20/26 (76.9)	18/44 (40.9)	**0.03**	21/26 (80.8)
Mild, n (%)	23/70 (32.9)	9/26 (34.6)	14/44 (31.8)	1.00	
Moderate to severe, n (%)	15/70 (21.4)	11/26 (42.3)	4/44 (9.1)	**0.01**	
ADHD, n (%)	21/74 (28.4)	7/26 (26.9)	14/48 (29.2)		Reported in four patients
ASD, n (%)	18/74 (24.3)	6/26 (23.0)	12/48 (25.0)		Reported in six patients
Motor impairment, n (%)	1/75 (1.3)	1/26 (3.8)	0/49 (−)	1.00	Not reported
Language impairment, n (%)	20/71 (28.2)	10/25 (40.0)	10/46 (21.7)	1.00	Reported in four patients
Brain imaging anomalies, n (%)	14/46 (30.4)	10/21 (47.6)	5/16 (31.3)	1.00	9/25 (36.0)
**KBG specific characteristics**					
Fulfilling Skjei/Low criteria, n (%)^b^	56/60 (93.3)	21/22 (95.5)	35/38 (92.2)		30/31 (96.8)
Macrodontia, n (%)^c^	48/59 (81.4)	16/19 (84.2)	32/40 (80.0)		23/29 (79.3)
Characteristic facial appearance, n (%)^d^	64/73 (87.7)	23/25 (92.0)	41/48 (85.4)		30/33 (90.9)
Postnatal short stature, n (%)^e^	30/71 (42.3)	12/23 (52.2)	18/48 (37.5)		12/35 (34.3)
Hand anomalies, n (%)^f^	51/67 (76.1)	16/21 (76.2)	35/46 (76.1)		24/34 (70.6)
Costovertebral anomalies, n (%)^g^	13/24 (54.2)	5/8 (62.5)	8/16 (50.0)		8/24 (33.3)
Delayed bone age, n (%)^h^	6/17 (35.3)	1/4 (25.0)	5/13 (38.5)		5/19 (26.3)

*Note*: Values represent the number of cases in which a feature is present (numerator) and the number of cases in which the presence of this feature was known (denominator). Percentages are displayed in parentheses. Significance is tested using the chi‐squared test or Fisher's exact test one‐sided when indicated. Bold indicates significant values (*P* 〈 0.05).

Abbreviations: ADHD, attention deficit hyperactivity disorder; ASD, autism spectrum disorder; ID, intellectual disability.

^a^ Cognition, based on intellectual quotient or level of functioning: Normal: IQ > 80, Mild ID: IQ 55–70, Moderate ID: IQ 40–55, Severe ID: IQ <40.^31^

^b^ Skjei and Low criteria: “fulfilling” is documented when a patient met at least one of both clinical criteria.

^c^ Macrodontia: mesiodistal width of central incisors ≥10 mm in male and 9.7 mm in females.

^d^ Characteristic facial appearance: presence of at least one representative finding from at least three of the following six categories: (1) craniofacial shape; (2) hirsutism; (3) eyes; (4) ears; (5) nose; (6) mouth.

^e^ Postnatal short stature: height ≤3rd centile with birth length, if known, >3rd centile.

^f^ Hand anomalies: fifth finger clinodactyly, clinical brachydactyly, and/or short tubular bones on radiographic examination.

^g^ Costovertebral anomalies: abnormal curvature of the spine, cervical ribs, and/or vertebral/endplate anomalies.

^h^ Delayed bone age: <2SD.

### 3.3 Phenotypes in the epilepsy vs the non‐epilepsy group

The majority of both patients with and without epilepsy had DD (92.3% and 83.7%, respectively) and the prevalence of ASD and ADHD was similar between these groups. Developmental plateauing or regression was only reported in two patients with epilepsy (10.5%, n = 2/19) and was related to the onset (n = 1; not further specified) or frequency (n = 1; loss of speech) of seizures. Intellectual disability was more frequently seen in the epilepsy group compared with the non‐epilepsy group (respectively 76.9% vs 40.9%, RR 1.9 [95% CI 1.2–2.8]) and was more often moderate to severe in those with versus those without epilepsy (respectively 42.3% vs 9.1%, RR 4.6 (95% CI 1.7–13.1). Language impairment was also more frequently observed in those with (n = 10/25) versus without epilepsy (n = 10/46), but differences were not significant (RR 1.6 [95% CI 0.7–3.4]; Table 1).

### 3.4 Epilepsy characteristics (n = 26)

#### 3.4.1 Seizure types

The median age at seizure onset was 4 years with a range from 12 months to 22 years (Table 2). At onset, 15 patients (57.7%) had generalized seizures; five (19.2%) had focal seizures and in six patients (23.1%) the seizure type at onset was unknown. The most common generalized seizure type at onset was tonic–clonic (n = 6), followed by absences (n = 4), myoclonic (n = 2), and myoclonic‐atonic seizures (n = 2). Nine patients (34.6%) had multiple seizure types. Three patients with generalized seizures at onset later developed focal seizures, but no patients with focal seizures at onset developed additional generalized seizure types. Over time, generalized seizures were more often seen than focal seizures (57.7% versus 34.6%, respectively, see Table 2). Reported triggers for seizures were fever (n = 4), stress (n = 2), hyperventilation (n = 1), and agitation (n = 1).

**TABLE 2:** Epilepsy phenotype in patients with KBG syndrome.

	Epilepsy group of patient cohort (n = 26)	Historical cohort (n = 37)
**Median age at onset seizures (range)**	4 year (12 month – 20 year)	3 year (6 days – 8 year, known in 20)
**Seizure type at onset**		
Generalized, n (%)	15/26 (57.7)	7/37 (18.9)
Focal, n (%)	5/26 (19.2)	7/37 (18.9)
Unknown, n (%)	6/26 (23.1)	24/37 (64.9)
**All documented seizure types**		
Generalized seizures, n (%)	15/26 (57.7)	7/37 (18.9)
Tonic–clonic	7/26	4/37
Absences	4/26	1/37
Myoclonic	4/26	0/37
Myoclonic‐atonic	2/26	1/37
Unclassified drop attack	2/26	0/37
Atypical absences	1/26	0/37
Tonic	1/26	0/37
Spasms	1/26	0/37
Myoclonic absences	0/26	1/37
Focal seizures, n (%)	8/26 (30.8)	8/37 (21.6)
Focal to bilateral tonic–clonic	5/26	4/37
Focal with impaired awareness	3/26	4/37
Focal non‐motor seizures	1/26	1/37
Focal motor seizures	0/26	1/37
Focal clonic	0/26	1/37
Unknown onset seizures, n (%)	7/26 (23.9)	9/37 (24.3)
Tonic–clonic	4/26	4/37
Non‐motor	3/26	3/37
Atonic	2/26	1/37
Clonic	1/26	1/37
Myoclonic	0/26	2/37
Tonic	0/26	1/37
Motor	0/26	1/37
No information available, n (%)	0/26 (0)	13/37 (35.1)
Febrile seizures	Reported in one patient	Not reported
Status epilepticus	Reported in two patients	Reported in one patient
**Multiple seizure types**	9/25 (36.0)	Unknown
**EEG results**		
Focal epileptic discharges, n (%)	7/23 (30.4)	Reported in six patients
Generalized epileptic discharges, n (%)	5/23 (21.7)	Reported in two patients
Generalized & focal epileptic discharges, n (%)	6/23 (26.1)	Reported in one patient
Unclassified epileptic discharges, n (%)	2/23 (8.7)	Not reported
No epileptiform discharges, n (%)	3/23 (13.0)	Reported in one patient
**Epilepsy type**		
Generalized, n (%)	9/22 (40.9)	6/16 (37.5)
Combined generalized and focal, n (%)	9/22 (40.9)	2/16 (12.5)
Focal, n (%)	4/22 (18.2)	8/16 (50.0)
**Seizure frequency**		
Daily	11/26	Unknown
Weekly	3/26	Unknown
Monthly	4/26	Unknown
<1/Month	4/26	Unknown
Unknown	4/26	Unknown
**Seizure remission**		
Clinical remission^a^, n (%)	12/23 (52.2)	Reported in nine patients
Median age at remission (range)	5 years (1 year 9 months‐23 years)	8 years 9 months (21 days‐14 years, known in six patients)
Number of ASM used at time of remission, median (range)	1 AED (1–6)	Unknown
**Prescribed anti‐seizure medications**		
Valproic acid	15/21	7/15
Lamotrigine	5/21	6/15
Carbamazepine	6/22	1/15
Levetiracetam	4/21	5/15
Clobazam	3/21	2/15
Phenobarbital	2/21	1/15
Ethosuximide	2/21	1/15
Oxcarbazepine	2/21	2/15
Rufinamide	1/21	0/15
Felbamate	1/21	0/15
Phenytoin	2/21	0/15
Topiramate	2/21	4/15
Zonisamide	0/21	1/15
Diazepam	0/21	1/15
Cannabidiol	0/21	1/15

*Note*: Values represent the number of cases in which a feature is present (numerator) and the number of cases in which the presence of the feature was known (denominator).

Abbreviations: AED, antiepileptic drugs, ID, intellectual disability.

^a^ Clinical remission is defined as 1 year without seizures.

#### 3.4.2 Epilepsy type

The epilepsy type was more often generalized (40.9%) or combined generalized and focal (40.9%) compared with focal (18.2%). Patients with a generalized or combined epilepsy type often had daily seizures (n = 7/9 and n = 5/9 patients, respectively), while all four patients with focal epilepsy had weekly or less frequent seizures. No association was seen between epilepsy type and age at seizure onset.

#### 3.4.3 Neurodevelopment

ID was present in 4/4 patients with focal epilepsy, in 7/9 with a combined, and 5/9 with a generalized epilepsy type, but was more often moderate to severe in those with a combined (n = 4/7) or generalized (n = 4/5) versus a focal epilepsy (n = 1/4). The prevalence of language impairment was similar between patients with different epilepsy types (ranging from 44% to 50%), but three non‐verbal patients all had generalized epilepsy. Early seizure onset was, not significantly, correlated with more severe ID: 3/4 patients (75%) with seizure onset during infancy (<2 years of age) developed moderate to severe ID, compared with 5/13 (38%) with seizure onset in childhood (2–10 years of age) and 2/7 patients (29%) with seizure onset in adolescence to adulthood (>10 years of age).

#### 3.4.4 Epilepsy syndrome

The epilepsy characteristics of the patients varied, ranging from two episodes with seizures in two patients to a severe developmental and epileptic encephalopathy (DEE) in three other patients. Eight patients had a phenotype overlapping with known epilepsy syndromes: Lennox–Gastaut syndrome (p3 and p69), myoclonic epilepsy in infancy (p20 and p39), epilepsy with myoclonic‐atonic seizures (p4 and p75), and childhood absence epilepsy (p52 and p58).

#### 3.4.5 Epilepsy treatment and outcome

At time of last assessment, 12/23 (52.1%) patients were seizure free, with seizure offset at a median age of 5 years (range 1 year and 9 months – 23 years). In two other patients, only a temporary remission was seen after starting ASM; seizures recurred after 2 and 4 years, respectively. All four patients with focal epilepsy had seizure remission compared with 7/9 patients with generalized epilepsy and 3/9 with a combined generalized and focal epilepsy. Overall, 12 different ASM were administered: valproic acid was most commonly prescribed (n = 16), followed by lamotrigine (n = 6), carbamazepine (n = 5), and levetiracetam (n = 3) (Table 2). In total, 12/23 patients (52.2%) used a single ASM; 5/23 (21.7%) were prescribed two; and 6/23 (26.1%) more than two. Seven patients had drug‐resistant epilepsy, of whom six had moderate to severe ID and four (known in 6) had seizure onset <3 years of age. One of them eventually reached seizure remission 7 years after seizure onset. Two patients started with a ketogenic diet after failure of multiple ASM, which was effective in one of them.

### 3.5 Epilepsy in the historical cohort (n = 37)

From the literature, we identified another 42 patients, who were not yet included in our cohort^4^, ^5^, ^6^, ^7^, ^9^, ^12^, ^13^, ^14^, ^15^, ^16^, ^17^, ^19^, ^33^, ^34^, ^35^, ^36^, ^37^, ^38^, ^39^, ^40^, ^41^ (Table S3). Of them, 37 patients were included: 34 had a truncating variant; two a missense variant; and one a partial deletion of *ANKRD11*. Five others had a 16q24.3 microdeletion including *ANKRD11* among other genes and are discussed separately. The clinical characteristics of patients in the historical cohort did not differ significantly from those in our epilepsy group (Table 1). In the historical cohort, there was a similar distribution of focal vs generalized seizures at onset (both 18.9%), but a focal epilepsy type was more common than a generalized epilepsy type (respectively 50.0% and 37.5%; Table 2). Focal epilepsy was more prevalent in the historical cohort, compared with our study cohort (50.0% vs 18.2%, respectively). Four patients had overlap with specific epilepsy syndrome diagnoses, including genetic epilepsy with febrile seizures+ (GEFS+, n = 1); epilepsy with myoclonic‐atonic seizures (EMAtS, n = 1); epilepsy with myoclonic absences (n = 1); and Lennox–Gastaut syndrome (n = 1). The epilepsy of patient 4 showed overlap with both GEFS+ and EMAtS, but she and her family members also had a variant in *SCN9A*, which might have contributed to the epilepsy phenotype, according to the authors. Seizure freedom was reported for nine patients and three patients had drug‐resistant epilepsy. Seizure freedom for unknown period and nonspecific descriptions as “well response to antiepileptic drugs” and “seizure reduction after initiating treatment” were mentioned in 10 other patients.

### 3.6 Genotype–phenotype correlation

**FIGURE 1:**
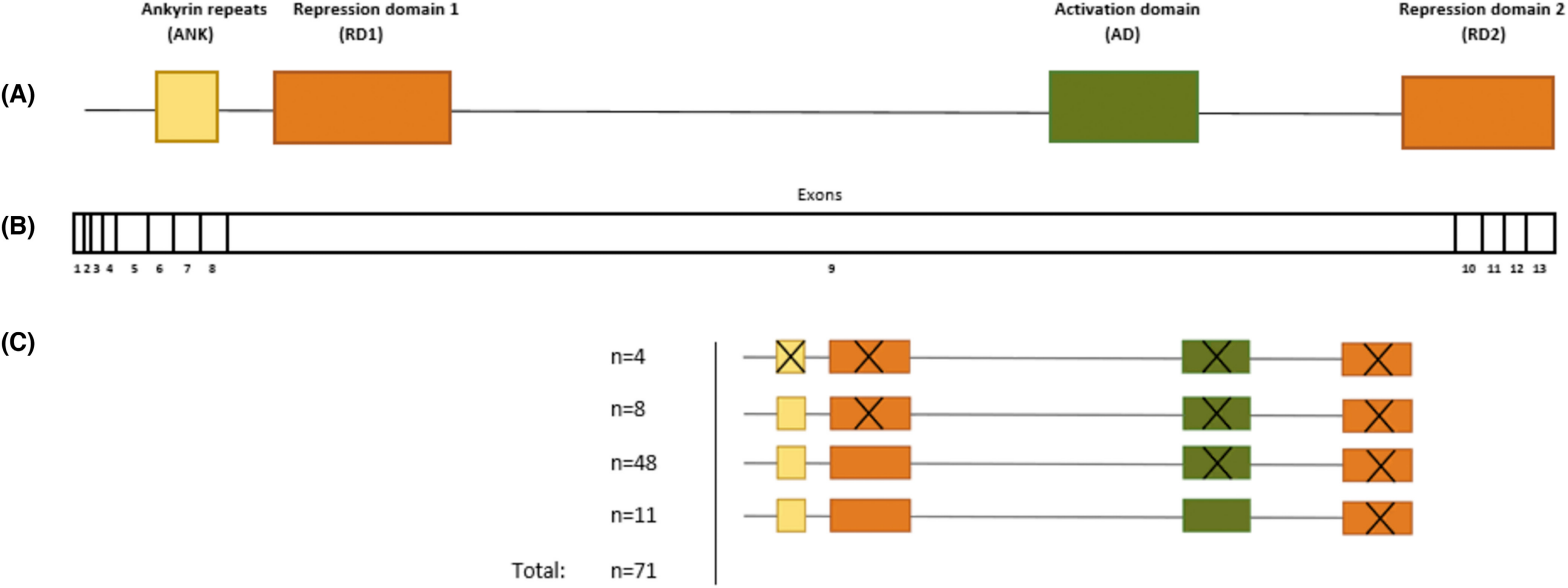
*ANKRD11* truncating variants in different exons and functional domains. (A) Schematic representation of the localization of ANKRD11 functional domains. (B) Schematic representation of the 13 different exons of *ANKRD11*. (C) Schematic representation of the distribution of patients with truncating variants and their disrupted functional domains.

Almost all truncating variants, both in patients with and without epilepsy, were located in exon 9 (91.5%, n = 65 see Table S1), the largest exon of *ANKRD11*. No correlation was found between the presence of epilepsy or specific epilepsy characteristics (type of epilepsy; seizure freedom; and drug‐resistant epilepsy) and the number of disrupted functional domains in patients with a truncating variant (Figure 1; data not shown). Seven patients with epilepsy had an *ANKRD11* variant that was identical to that of other patients in our cohort who did not have epilepsy. In one family, three patients (p34, p35, and p36) had an identical *ANKRD11* nonsense variant, but epilepsy was only present in two of them.

### 3.7 Epilepsy in patients with a polygenic 16q24.3 microdeletion

In total, eight patients had a chromosome 16q24.3 microdeletion including *ANKRD11* among other genes, of whom six had epilepsy (Table 3 and Table S4). Both focal and generalized seizure types and epilepsy types were seen. Patient 80 had a DEE overlapping with Lennox–Gastaut syndrome. These patients with polygenic microdeletions and epilepsy more often had moderate to severe cognitive impairment compared with patients with truncating variants and epilepsy (respectively 3/4, 75% vs 11/26, 42.3%; *P* = 0.32).

**TABLE 3:** Genotype and phenotypes in patients with a 16q24.3 microdeletion and epilepsy.

Patient ID	Publication	Position (size) (*GRCh37/hg19*)	Deleted genes	Epilepsy characteristics	Developmental delay	Cognition	Motor/Language impairment
P80	This study	Chr16: g.(89 179 707‐89 435 515) del (260 Kb)	ANKRD11 (exon 3–13), ZNF778, SLC22A31, CHD15, ACSF3	Seizures: Tonic–clonic and tonic seizures since 2 years of age, status epilepticus	Yes, including developmental regression at 2 years of age in speech, walking, and controlled urinating	Severe ID	Language and motor impairment
				EEG: multifocal epileptic discharges			
				Epilepsy type: focal			
				Epilepsy syndrome: DEE			
				Prognosis: Inadequate effect of 9 ASM			
L‐P38	Novara et al. (2017)	Chr16: g.(88630607_89,607 742)del (977 Kb)	SPG7, ANKRD11 (completely), ZNF778, SLC22A31, CHD15, ACSF3, CBFA2T3, PABPN1L, TRAPPC2L, GALNS, APRT, CDT1, PIEZO1, CTU2, RNF166, SNAI3, MVD, CYBA, IL17C, ZC3H18	Seizures: Generalized tonic–clonic seizures with onset at 15 years of age EEG: multifocal epileptic discharges Epilepsy type: combined generalized and focal Prognosis: Seizure offset at 23 Years of age	Yes	Normal	Language impairment
L‐P39	Novara et al. (2017) & Goldenberg et al. (2016)	chr16:g.(89161684_89,505 106)del (343 Kb)	ANKRD11 (exons 2–13), ZNF778, SLC22A31, CHD15, ACSF3	Seizures: generalized seizures with onset at 1 year of age EEG: Epilepsy type: – Epilepsy syndrome: – Prognosis: drug‐resistant seizures	Yes	ID (unknown degree)	Language and motor impairment
L‐P40	Ockeloen et al. (2014)	Chr16: g.(88231090_89,388 103)del (1.16 Mb)	ANKRD11, ZNF778, SLC22A31, CHD15, ACSF3, CBFA2T3, PABPN1L, TRAPPC2L, GALNS, APRT, CDT1, PIEZO1, CTU2, RNF166, SNAI3, MVD, CYBA, IL17C, ZC3H18, ZFPM1, ZNF469	Seizures: atypical seizures with onset at 3 months of age EEG: – Epilepsy type: – Epilepsy syndrome: – Prognosis: –	Yes	Unknown	Unknown
L‐P41	Willemsen et al. (2010)	Chr16: g.(89.12 Mb – 89.50 Mb)del (378 Kb)	ANKRD11 (exons 2–13, ZNF778, SLC22A31, CDH15, ACSF3	Seizures: Generalized absences and other generalized seizures with onset at 3 years of age	Yes, including motor impairment	Moderate ID	Language and motor impairment
				EEG: – Epilepsy type: generalized epilepsy Epilepsy syndrome: – Prognosis: –			
L‐P42	Willemsen et al. (2010)	Chr16: g.(87.50 Mb – 89.60 Mb)del (2.07 Mb)	SPG7, ANKRD11 (completely), CDH15, ZNF778, SLC22A31, CHD15, ACSF3, CBFA2T3, PABPN1L, TRAPPC2L, GALNS, APRT, CDT1, PIEZO1, CTU2, RNF166, SNAI3, MVD, CYBA, IL17C, ZC3H18, ZFPM1, ZNF469, BANP, CA5A, CLC7A5, KLHDC4, JPH3, ZCCHC14	Seizures: Focal seizures with impaired Awareness EEG: focal epileptic discharges Epilepsy type: focal epilepsy Epilepsy syndrome: – Prognosis: –	Yes	Moderate ID	Motor impairment

Abbreviations: ASM, anti‐seizure medication; EEG, electroencephalogram; ID, intellectual disability; L, literature.

## 4 DISCUSSION

We present an original cohort of 75 patients from nine European and North‐American medical centers with genetically confirmed KBG syndrome, of whom one‐third had epilepsy. To date, this is the largest study on epilepsy in KBG syndrome. We studied the epilepsy phenotypes in these 26 patients, supplemented with 37 previously reported patients. We found that epilepsy in KBG syndrome typically had its onset in early childhood. The majority had generalized seizures at onset with tonic–clonic seizures being most common. The epilepsy type was mostly classified as generalized or combined generalized and focal, although focal epilepsy was more commonly reported in the historical cohort. There is no electroclinical syndrome specific for KBG syndrome, but overlap has been seen with other (combined focal and) generalized epilepsy syndromes, including Lennox–Gastaut syndrome, epilepsy with myoclonic–atonic seizures, myoclonic epilepsy in infancy, childhood absence epilepsy, and epilepsy with myoclonic absences. Most patients with epilepsy reached seizure remission on ASM, but a quarter of patients had drug‐resistant epilepsy.

In their recent paper, Auconi and colleagues also report a variable epileptic phenotype with similar seizure types (tonic–clonic, focal to bilateral tonic–clonic; myoclonic) but no syndrome‐specific EEG pattern in a cohort of 11 patients with KBG syndrome and epilepsy.^8^ However, a combined focal and generalized epilepsy type was not reported, in contrast to our study. Auconi et al. report seizure remission in all patients during follow‐up (seizure offset after 15 days ‐ 6 years [range]), indicating the self‐limiting course of epilepsy in KBG syndrome in most patients. They also reported two patients with a DEE, complementing our findings, indicating that the epilepsy in KBG syndrome can be severe in a minority of patients. Our study also describes patients with KBG syndrome and epilepsy with ongoing seizures at time of assessment, of which five patients had seizure activity for at least 10 years despite treatment with ASM.

We showed that a diagnosis of epilepsy in patients with KBG syndrome was correlated with a poorer neurodevelopmental outcome. Patients with KBG syndrome and epilepsy more often had ID that was also more severe than in those without epilepsy. Patients with epilepsy also had more frequently language impairment, although this difference was not statistically significant, possibly due to low numbers. Furthermore, two patients had developmental regression related to the onset or frequency of seizures. These results highlight that epilepsy is a striking feature of KBG syndrome and seems to be associated with poorer neurodevelopmental outcome in some patients. We do not recommend to routinely perform EEGs in patients who do not have clinical seizures, because a normal interictal EEG does not exclude an epilepsy diagnosis and the clinical significance of finding epileptic EEG discharges in patients without clinical seizures is often uncertain.

KBG syndrome is similar to other developmental encephalopathies of genetic etiology, such as Angelman syndrome, Mowat–Wilson syndrome, Rett syndrome, and Pitt–Hopkins syndrome, since they all share a spectrum of phenotypic characteristics such as facial dysmorphism, neurodevelopmental delay, ID, congenital anomalies and epilepsy. All these syndromes show variability with respect to the epilepsy characteristics present in affected patients.^42^, ^43^, ^44^, ^45^ In Rett syndrome, the severity of epilepsy is also an important contributor to clinical severity.^42^ For the other syndromes, this relation is unknown.

No genotype–phenotype correlation was identified for the presence of epilepsy or epilepsy characteristics in relation to the number of disrupted functional domains in our cohort. Li et al. previously suggested that ID was significantly more severe in those with pathogenic truncating variants disrupting three functional domains (AD, RD1, and RD2) or the RD2 domain alone.^20^ Also, they showed that patients with pathogenic truncating variants retaining RD1 and AD had more severe ID than retaining RD1 alone. These findings can only be understood when these truncating variants do not—or only partially—result in nonsense‐mediated decay. Walz et al. showed that truncating *ANKRD11* variants can lead to premature termination codons, resulting in proteins that lack the degradation signal at the C‐terminus D‐box.^32^ Furthermore, Sanger sequencing of cDNA obtained from a blood mRNA sample of a patient with a truncating variant revealed the presence of the mutant allele, suggesting that a complete nonsense‐mediated mRNA decay did not occur.^32^ In this study, we did not find a genotype–phenotype correlation, possibly due to the lack of power. Probably, other factors—beyond the *ANKRD11* gene—also account for the phenotypic heterogeneity, such as the function of other (transcription regulating) genes or environmental factors. Patients with a polygenic chromosome 16q24.3 microdeletion including *ANKRD11* and epilepsy had a poorer cognitive outcome in comparison with patients with truncating variants and epilepsy, but findings were not significant, possibly due to low numbers. This finding could be explained due to deletions of other possibly important genes for neurodevelopment (eg, *ZNF778* ^38^).

Our study has two major limitations, due to its retrospective design. First, a selection bias could have influenced our number of patients with epilepsy among those with KBG syndrome, since patients with a diagnosis of epilepsy might be more willing to consent for participating in this epilepsy study, or be more likely to be seen in reporting centers. This bias may have resulted in a higher prevalence of patients with epilepsy or more severe epilepsy phenotypes. Second, for some patients, data regarding the epilepsy phenotype — especially EEG results — were missing. To minimize missing data, we contacted the participating medical centers or the original authors of previously reported patients for additional data. Nevertheless, for many patients the epilepsy classifications (according to ILAE standards^23^, ^24^, ^25^, ^26^, ^27^, ^28^) were categorized in the “unknown” category due to missing information.

To conclude, epilepsy is a prominent feature of KBG syndrome and presents with a heterogeneous epilepsy phenotype, but most often with a generalized or a combined generalized and focal epilepsy with onset in childhood. In a significant proportion of KBG syndrome patients with epilepsy, seizures are drug‐resistant, which was only rarely reported in previous literature. Moreover, epilepsy in patients with KBG syndrome is associated with poorer developmental outcome. Because some patients with KBG syndrome may come to medical attention because of their seizures, it is important that KBG syndrome is recognized as a causal etiology. Subsequently, healthcare professionals who take care of patients with KBG syndrome should pay attention to the possible occurrence of seizures.

## AUTHOR CONTRIBUTIONS

N. Buijsse, F.E. Jansen, E.H. Brilstra, and D.R.M. Vlaskamp designed the study, analyzed the data, and wrote the manuscript. All authors contributed to data collection and manuscript editing.

## FUNDING INFORMATION

This research received no specific grant from any funding agency. S.Sisodiya was supported by the Epilepsy Society.

## CONFLICT OF INTEREST STATEMENT

The authors report no financial disclosures or competing interests relevant to this study.

## ETHICS STATEMENT

This study has been reviewed and approved by the Medical Ethical Committee of the University Medical Centre Utrecht (UMCU) and does not fall under the Medical Research Involving Human Subjects Act (reference: 20‐013/C). We confirm that we have read the Journal's position on issues involved in ethical publication and affirm that this report is consistent with those guidelines.

## Supporting information


Figure S1

Table S1

Table S2

Table S3

Table S4

## Data Availability

The authors confirm that the data supporting the findings of this study are available within the article and its supplementary materials.
