# Supplementary figures and images for: Epilepsy is an important feature of KBG syndrome associated with poorer developmental outcome
Source: Epilepsia Open. 2023 Aug 18;8(4):1300–13. doi: 10.1002/epi4.12799 (PMC10690702; doi:10.1002/epi4.12799)

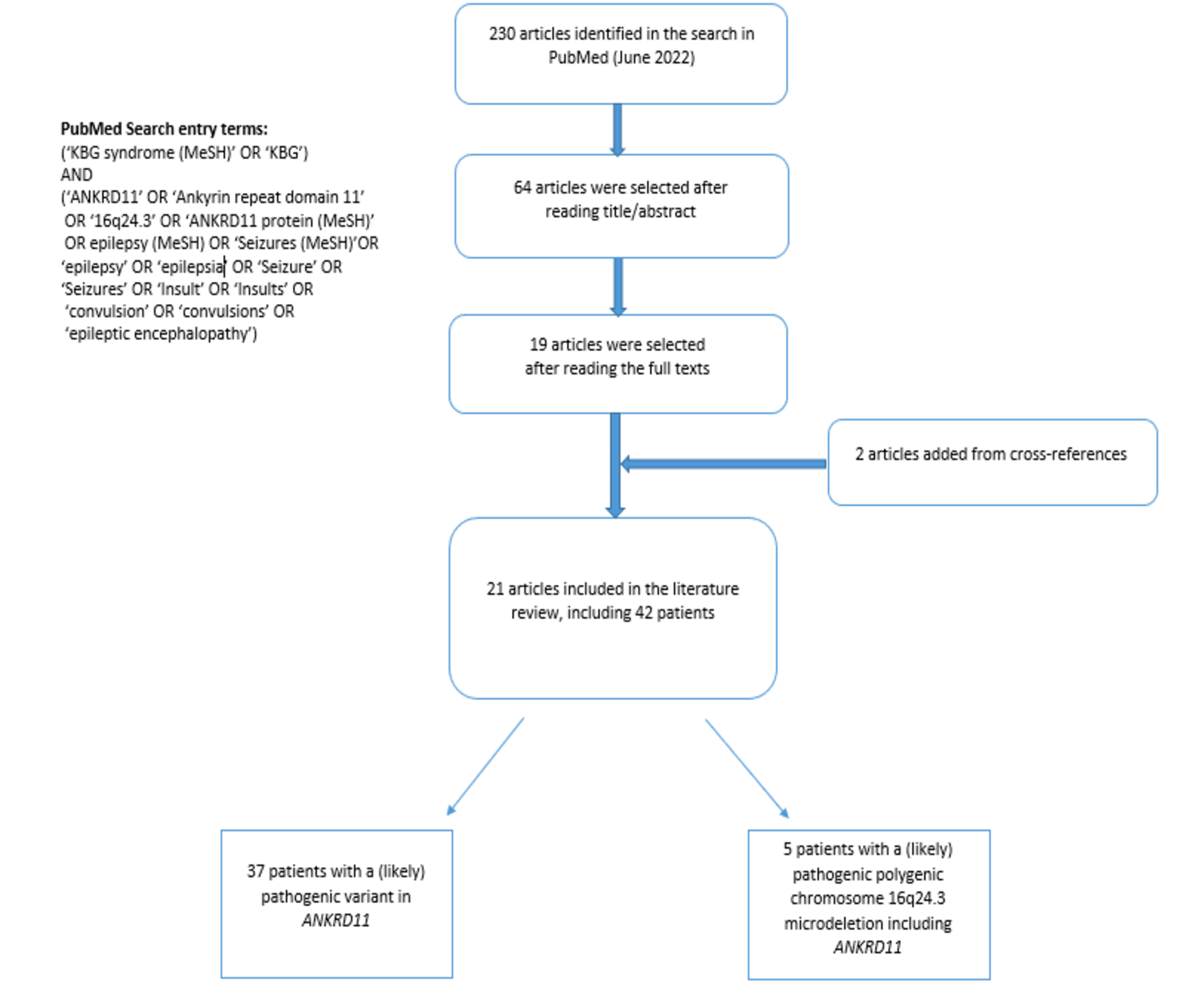

Supplement: Supplementary file 1 — Figure S1 [file EPI4-8-1300-s001.tiff]
